# Metformin Ameliorates Lipopolysaccharide-Induced Depressive-Like Behaviors and Abnormal Glutamatergic Transmission

**DOI:** 10.3390/biology9110359

**Published:** 2020-10-26

**Authors:** Jiang Chen, Tian Zhou, A-Min Guo, Wen-Bing Chen, Dong Lin, Zi-Yang Liu, Er-Kang Fei

**Affiliations:** 1 Laboratory of Synaptic Development and Plasticity, Institute of Life Science, Nanchang University, Nanchang 330031, China; ncuchenjiang@163.com (J.C.); gam1204@163.com (A-M.G.); cwb100405@163.com (W.-B.C.); lk1073216919@163.com (D.L.); nculzy2020@163.com (Z.-Y.L.); 2 Department of Biological Science, School of Life Sciences, Nanchang University, Nanchang 330031, China; 3 School of Basic Medical Sciences, Nanchang University, Nanchang 330031, China; zhoutian@ncu.edu.cn

**Keywords:** depression, metformin, lipopolysaccharide (LPS), synaptic transmission

## Abstract

**Simple Summary:**

Metformin is a promising drug for diabetes and has been reported to have antidepressant effects in depression patients or patients with comorbid depression and other diseases. However, it is largely unclear how metformin ameliorates depressive-like behaviors. To this end, we injected mice with a bacterial endotoxin (lipopolysaccharide) to induce depressive-like behaviors such as increased immobility in the forced swimming test and tail suspension test. In this depression mouse model, metformin administration ameliorated depressive-like behaviors. Glutamate is a major excitatory signal for the communications between neurons in the brain. Dysfunction of glutamatergic neurotransmission is implicated in the pathogenesis of depression. Glutamatergic transmission was elevated in our depression mouse model. Metformin administration also recovered the glutamatergic transmission deficit in the model. Taken together, our results suggest metformin had antidepressant effects and can correct abnormal glutamatergic transmission in the lipopolysaccharide-induced depression mouse model. These findings provide new insights into the underlying mechanism by which metformin acts against depression.

**Abstract:**

Metformin, a first-line drug for type 2 diabetes mellitus (T2DM), has been found to reduce depressive symptoms in patients with comorbid depression and other diseases. However, it is largely unclear how metformin ameliorates depressive-like behaviors. Here, we used lipopolysaccharide (LPS) to induce depressive-like behaviors in mice and found that LPS-treated mice exhibited increased immobility in the forced swimming test (FST) and tail suspension test (TST), as well as increased glutamatergic transmission. Furthermore, metformin administration in the LPS-treated mice ameliorated depressive-like behaviors and elevated glutamatergic transmission. Our results suggest that metformin has antidepressant effects and can correct abnormal glutamatergic transmission, providing an insight into the underlying mechanism by which metformin acts against depression.

## 1. Introduction

Depression is a common and disabling mental illness with an estimated lifetime prevalence of one in six people [1] and has been identified as one of the leading contributors to the global health burden [2]. Depression is characterized by a persistent low mood, loss of interest in normally enjoyable activities, reduced self-esteem, insomnia and suicide [3]. Current antidepressant medications or treatments are associated with limited symptomatic efficacy and delayed responses [4]. Meanwhile, depression and diabetes are interrelated. The risk for depression is double in type 2 diabetes mellitus (T2DM) patients [5,6], implying that depression and T2DM may share biological pathways and treatments for T2DM may also alleviate depression.

The biguanide metformin is a first-line drug for T2DM therapy with a long history. Metformin lowers the plasma glucose level and enhances insulin sensitivity [7]. Metformin has been used in the treatment of antipsychotic-related metabolic syndrome [8,9]. Intriguingly, it has also been found that metformin may reduce depressive symptoms in patients with comorbid depression and other disorders [10,11]. A case study [10] reported that metformin and spironolactone (a mineralocorticoid receptor antagonist) treatments relieved depression in a young woman with depression and polycystic ovarian syndrome (PCOS). In another clinical study [11], 58 patients diagnosed with depression and T2DM were divided into two groups, with one receiving metformin and the other receiving placebo treatment for 24 weeks, and it was found that metformin improved depressive symptoms. Recently, metformin was also found to be effective as an adjunct to antidepressants in nondiabetic major depressive disorder (MDD) patients [12]. Moreover, metformin can ameliorate depressive-like behaviors in rodent models of T2DM with depression [13,14]. In a rat T2DM model induced by streptozotocin and nicotinamide, foot-shocks were administrated to model comorbid depression in diabetic rats. Metformin also decreased the immobility time during the forced swimming test (FST) in this rat model [13]. In mice fed with a high-fat diet, metformin reduced the immobility time in the tail suspension test (TST) [14]. Besides the mouse model of diabetes with comorbid depression, metformin is also reported to have antidepressant effects in depression mouse models [15,16]. In mice subjected to chronic social defeat stress (CSDS), metformin treatment rescued the depressive-like behaviors in social interaction behavioral test, sucrose preference test, FST and TST [15]. Metformin also alleviated depressive-like behaviors in the sucrose preference test and FST in a chronic unpredictable mild stress (CUMS) mouse model [16]. These studies suggest metformin may exert antidepressant effects. However, it is largely unclear how metformin ameliorates depressive-like behaviors.

Several studies have reported that dysfunction of serotonergic transmission plays roles in the pathogenesis and pathophysiology of depression and metformin can stimulate serotoninergic neuron excitability and serotonergic transmission [14,17]. Combined metformin and fluoxetine (a selective serotonin reuptake inhibitor) treatment promotes the antidepressant efficacy [18]. Meanwhile, glutamate is the major excitatory neurotransmitter released at synapses in the brain. Dysfunction of glutamatergic neurotransmission is implicated in the pathogenesis of depression [19,20,21]. It has been reported that glutamate levels are increased in the serum, cerebrospinal fluid and brain tissues of patients with depression [22,23,24]. Altered expression or function of glutamate N-methyl-d-aspartate receptor (NMDAR) subunits is also found in depression patients [25,26,27,28,29]. In rodent stress models of depression, acute stress increases glutamate release, while chronic stress leads to neuronal atrophy in the prefrontal cortex and hippocampus and alters glutamatergic transmission [30,31,32,33]. However, decreased glutamate was also observed in depression [34]. Ketamine, a novel antidepressant, is found to increase glutamate release [35]. These findings suggest that not only serotoninergic transmission but also glutamatergic transmission plays important roles in depression.

Lipopolysaccharide (LPS), a bacterial endotoxin, has been reported to produce depressive-like behaviors in rodents, including increased immobility in FST and TST [36,37]. Here, we used LPS to induce depressive-like behaviors and detected the glutamatergic transmission in mice. Then, we examined whether metformin ameliorates depressive-like behaviors and affects the glutamatergic transmission in the LPS-induced depression mouse model. 

## 2. Materials and Methods 

### 2.1. Animals and Drug Treatments

Male C57BL/6 mice, 6–7 weeks old, purchased from the model animal research center of Nanjing University (Nanjing, China) were used in the experiments. All mice were housed in a constant temperature and humidity chamber at 25 °C, and sufficient food and water were administered daily. No more than 5 adult mice per cage were subjected to a 12-h light/dark cycle under standard conditions. All the mice were guaranteed to be hygienic. The animal experiments were carried out following the “Guidelines for the Care and Use of Laboratory Animals” promulgated by Nanchang University.

LPS (Sigma-Aldrich, St. Louis, MO, USA) was dissolved in sterile, endotoxin-free 0.9% (m/v) sodium chloride solution and injected intraperitoneally (i.p.) into mice at a dosage of 0.5 mg/kg (body weight) to stimulate subclinical infection without causing significant inflammation or other significant damage to the animals [38]. After 10-day (once daily) administration of LPS, the mice were subjected to behavioral tests and electrophysiological or other experiments.

Metformin (Sigma-Aldrich, St. Louis, MO, USA) was dissolved in 0.9% (m/v) NaCl solution (vehicle) and i.p. injected into mice at a dosage of 200 mg/kg (body weight) based on previous studies [39]. After i.p. injection of LPS, or saline, for 10 days and behavioral tests (FST and TST), the mice were then i.p. injected with 200 mg/kg metformin, or its vehicle, daily for another 10 days before other behavioral and electrophysiological tests or other experiments.

This study was approved by the Research Ethics Committee of Nanchang University on 9 March 2017 (Nanchang, China, NSFC 31771142).

### 2.2. Behavioral Tests

For the forced swim test (FST), mice were individually placed in a 2-L beaker containing clean water (23–25 °C). The depth of water was set as 20 cm to prevent the animal from touching the bottom with the tail or hind legs. Mouse behaviors were videotaped from the side for 6 min under red light. The immobility time of the last 4 min was recorded by an observer blinded to animal treatments. Immobility was defined as the duration that animals remained floating or stationary.

For the tail suspension test (TST), mice were suspended individually about 50 cm above the floor by fixing the tail with medical tape on the test box hook. Mouse behaviors were videotaped from the side for 6 min under red light. The last 4 min of test time was recorded by an observer blinded to animal treatment. Immobility was defined as the duration that animals did not struggle.

### 2.3. Electrophysiological Recording

Preparation of hippocampal slices was as described previously [40]. The mice were anesthetized with isoflurane, and the brain was quickly removed in ice-cold partial sucrose solution (PSS) containing 80 mM NaCl, 3.5 mM KCl, 4.5 mM MgSO_4_, 0.5 mM CaCl_2_, 1.25 mM NaH_2_PO_4_, 25 mM NaHCO_3_, 10 mM D-(+)-glucose and 90 mM sucrose. Coronal hippocampal slices (300 µm) were cut in ice-cold PSS using a VT-1000S vibratome (Leica, Wetzlar, Germany). Then, the hippocampal slices were transferred into traditional artificial cerebrospinal fluid (ACSF) containing 124 mM NaCl, 2.5 mM KCl, 2 mM MgSO_4_, 2.5 mM CaCl_2_, 1.25 mM NaH_2_PO_4_, 26 mM NaHCO_3_ ad 10 mM D-(+)-glucose and incubated for 30 min at 34 °C and 1 h at room temperature (25 ± 1 °C) before recording. All solutions contained saturated 95% O_2_/5% CO_2_ (vol/vol).

Slices were transferred into a recording chamber perfused with ACSF at a rate of 2–3 mL/min at 32–34 °C. CA1 pyramidal neurons were observed using an upright microscope equipped with an infrared-sensitive CCD camera (IR-1000, DAGE-MTI, Michigan City, IN, USA) and a 40× soaking lens (FN1, Nikon, Chiyoda, Japan). A borosilicate pipette was filled with an intracellular solution with a resistance of 3–5 MΩ. Whole-cell patch clamp recording was made using the Multiclamp 700B amplifier (Molecular Devices, Sunnyvale, CA, USA) with Digidata 1550A interface digitization and pClamp 10.6 software (Molecular Devices, Sunnyvale, CA, USA) acquisition at 3 kHz.

For mEPSC recording, 1 μM TTX and 20 μM bicuculline were added to ACSF to block action potential and inhibitory synaptic transmission, respectively. The pipettes were filled with potassium gluconate intracellular solution containing 130 mM K-gluconate, 5 mM NaCl, 1 mM MgCl_2_, 10 mM HEPES, 0.2 mM EGTA, 2 mM Mg-ATP and 0.1 mM Na-GTP (pH 7.30, 294 mOsm). The hippocampal CA1 pyramidal neurons were held at −70 mV and recorded by whole-cell patch clamp.

To detect the electric property of hippocampal CA1 pyramidal neurons, the pipettes were filled with potassium gluconate intracellular solution, the current clamp mode was performed and pulsed depolarization currents (from 0 to 350 pA at a step of 50 pA per 10 s) were injected. Furthermore, 20 μM bicuculline, 50 µM DL-AP5 and 20 µM DNQX were added to the ACSF to block inhibitory and excitatory synaptic transmission. 

For paired-pulse ratio recording, EPSCs were evoked by stimulating the Schaffer collaterals–CA1 pathway at a holding potential of –70 mV in the presence of 20 μM bicuculline, and the pipettes were filled with potassium gluconate intracellular solution. The interval of paired stimulations was set to 25, 50 and 100 ms. The ratio value was calculated as EPSC2/EPSC1 amplitudes.

### 2.4. Brain Morphological Analysis

Mice were anesthetized with isoflurane. After perfusion of 0.9% NaCl solution for 5–10 min, the mice were perfused with pre-cold 4% paraformaldehyde (PFA) for 5–10 min. Brains were weighed and fixed in 4% PFA at 4 °C overnight. Slices (50 µm) were cut in ice-cold PBS using a VT-1000S vibratome (Leica, Wetzlar, Germany). After washing with 0.01 M PBS, sections were attached to a gelatin-coated slide and air-dried at room temperature. Samples were incubated in a cresol purple solution and heated in a 60 °C water bath for 3–10 min. After washing 3 times with Milli-Q water, samples were put into 75%, 95% and 100% ethanol for 1 min in each. Finally, samples were immersed in xylene for 10 min, sealed with a neutral resin overnight and photographed with a fluorescence microscope (FN1, Nikon, Chiyoda, Japan).

### 2.5. Statistical Analysis

Before being analyzed, all data in our study were checked by the D’Agostino–Pearson omnibus normality test to prove they had a Gaussian distribution. The data were statistically analyzed and graphed using the GraphPad Prism software (San Diego, CA, USA). Electrophysiological data were analyzed using Clampfit and the Mini-Analysis software (Synaptosoft, Decatur, GA, USA). Images were edited using Photoshop (Adobe, San Jose, CA, USA). The experimental data were expressed as Min to Max or Mean ± SEM. Student’s t-tests were used to compare two groups of data. One-way ANOVA was used to compare three or more groups of data. Two-way ANOVA was used in electrophysiological studies when more than two parameters were analyzed. * *p* < 0.05; ** *p* < 0.01; *** *p* < 0.001.

## 3. Results

### 3.1. LPS Induces Depressive-Like Behaviors in Mice

**Figure 1:**
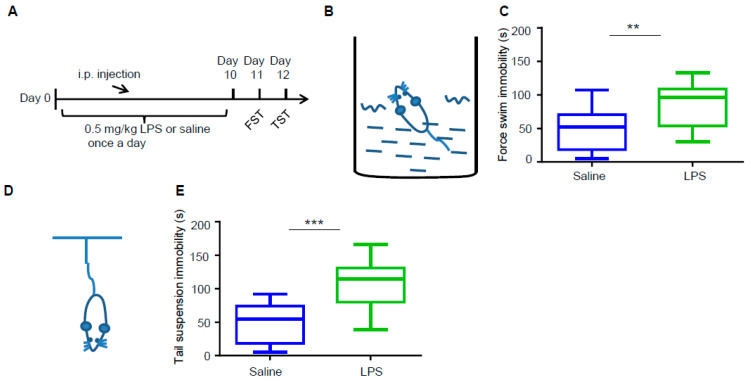
Lipopolysaccharide (LPS) induces depressive-like behaviors in mice. (**A**) Schematic illustration of the experimental procedure for LPS administration and behavioral tests for depressive-like behaviors in mice. C57BL/6 male mice at age of 6–7 weeks were intraperitoneally (i.p.) injected with 0.5 mg/kg LPS or an equal volume of saline daily for 10 days. The forced swimming test (FST) and tail suspension test (TST) were conducted at 24 and 48 h after the LPS treatment. After the behavioral tests, these mice were sacrificed for brain morphological analysis. (**B**,**C**) LPS treatment significantly increased the immobility time in FST. (**B**) Schematic diagram of mouse FST. (**C**) Quantification of the immobility time (s) in 4 min for each group of mice in FST. Data are shown as mean ± SEM (n = 14 mice for each group, Student’s *t*-test, ** *p* < 0.01). (**D**,**E**) LPS treatment significantly increased the immobility time in TST. (**D**) Schematic diagram of mouse TST. (**E**) Quantification of the immobility time (s) for each group of mice in TST. Data are shown as mean ± SEM (n = 14 mice for each group; Student’s *t*-test, *** *p* < 0.001).

To phenotype depressive-like behaviors in C57BL/6 mice, we intraperitoneally (i.p.) injected 0.5 mg/kg LPS into wild type (WT) male mice daily for 10 days (Figure 1A). Meanwhile, mice injected with the vehicle (saline) served as control. Compared with the control, LPS treatment did not alter mouse brain size or weight, nor did it alter the structure of the cortex or hippocampus (Figure S1). After 10 days of treatment, the mice were subjected to FST and TST sequentially in the next 2 days to confirm the depressive-like behaviors. As shown in Figure 1B–E, mice treated with LPS exhibited more immobility than control mice in both FST (Figure 1B,C) and TST (Figure 1D,E). These results indicate LPS induced depressive-like behaviors in our mouse model successfully. 

### 3.2. Miniature Excitatory Postsynaptic Current (mEPSC) Frequency of Hippocampal Pyramidal Neurons Is Increased in LPS-Induced Depression Mouse Model

**Figure 2:**
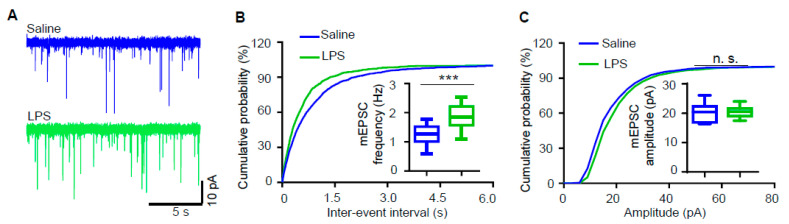
Miniature excitatory postsynaptic current (mEPSC) frequency of hippocampal pyramidal neurons is increased in the LPS-induced depression mouse model. (**A**) Representative traces of mEPSCs in CA1 pyramidal neurons from saline-treated (upper panel, blue) or LPS-treated (lower panel, green) mice. (**B**,**C**) Cumulative probability plots of mEPSC interevent intervals and histograms of mEPSC frequency (**B**) and amplitude (**C**) (n = 14 neurons from 3 saline-treated mice, n = 16 neurons from 3 LPS-treated mice; Student’s *t*-test, *** *p* < 0.001, n.s. *p* > 0.05).

To further detect whether LPS treatment affects the synaptic transmission, we recorded miniature excitatory postsynaptic currents (mEPSCs) in hippocampal CA1 pyramidal neurons. As shown in Figure 2, no difference was observed in mEPSC amplitudes between LPS-treated mice and control. However, mEPSC frequency was increased in mice treated with LPS (Figure 2A,B), which suggests that LPS treatment increases glutamatergic transmission. 

### 3.3. Metformin Ameliorates LPS-Induced Depressive-Like Behaviors

**Figure 3:**
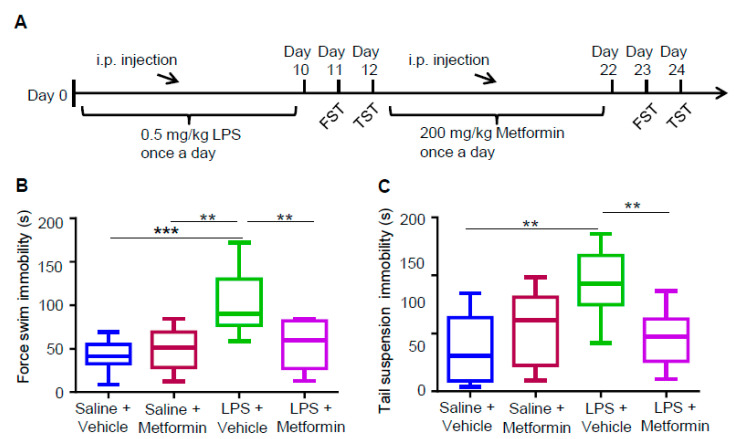
Metformin ameliorates LPS-induced depressive-like behaviors. (**A**) Schematic illustration of the experimental procedure for drug administrations and behavioral tests for depressive-like behaviors in mice. C57BL/6 male mice at age of 6–7 weeks were i.p. injected with 0.5 mg/kg LPS or an equal volume of saline daily for 10 days. After selection through TST and FST, the mice exhibited depressive-like behaviors were i.p. injected with 200 mg/kg metformin or an equal volume of its vehicle for another 10 days. Then FST and TST were conducted at 24 and 48 h after the metformin treatment. (**B**,**C**) Increased immobility time in FST and TST induced by LPS was reduced by metformin treatment. Quantification of the immobility time (s) in 4 min for each group mice in FST (**B**) and TST (**C**) (n = 8–9 mice for each group; one-way ANOVA, ** *p* < 0.005, *** *p* < 0.001).

To detect whether metformin can ameliorate LPS-induced depressive-like behaviors, we administrated the LPS-induced depression mouse model with metformin (Figure 3A). We divided these mice into four groups: (I) saline + vehicle, (II) saline + metformin, (III) LPS + vehicle and (IV) LPS + metformin. Brain size and weight, as well as the structure of the cortex and hippocampus, were observed, and no changes were found between the mice of these four groups (Figure S2). We also evaluated the depressive-like behaviors in these four groups with FST and TST. Like the results in Figure 1, treatment with LPS alone (group III vs. I) increased the immobility time in FST and TST (Figure 3B,C). However, metformin administration in LPS-treated mice (group IV vs. III) decreased the immobility time to that of group I (Figure 3B,C). These results suggest that metformin ameliorates LPS-induced depressive-like behaviors.

### 3.4. Metformin Reduces Increased mEPSC Frequency in LPS-Induced Depression Mouse Model 

**Figure 4:**
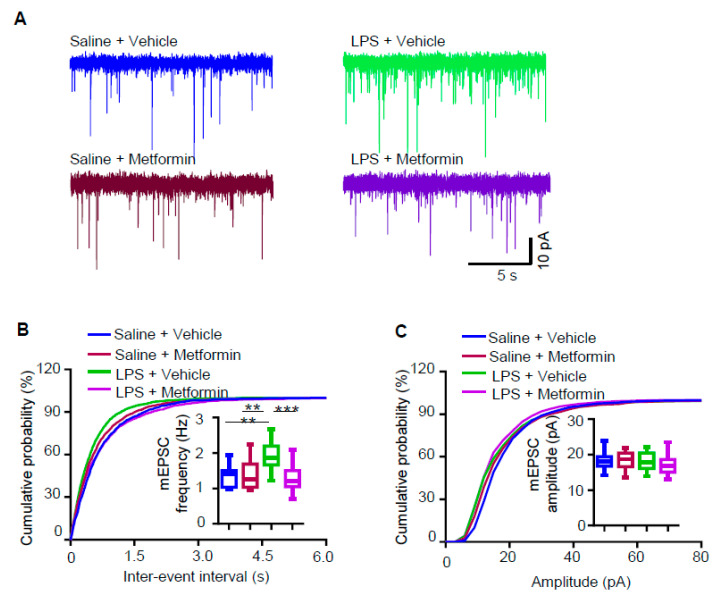
Metformin reduces increased mEPSC frequency in LPS-induced depression mouse model. (**A**) Representative traces of mEPSCs in CA1 pyramidal neurons from mice of four different groups: saline + vehicle (blue), saline + metformin (brown), LPS + vehicle (green) and LPS + metformin (purple). (**B**,**C**) Cumulative probability plots of mEPSC interevent intervals and histograms of mEPSC frequency (**B**) and amplitude (**C**) (n = 14–16 neurons from 3 mice for each group; one-way ANOVA, ** *p* < 0.005, *** *p* < 0.001).

mEPSC frequency was increased in LPS-induced depression mice, and metformin ameliorated LPS-induced depressive-like behaviors. We asked if metformin can reduce increased mEPSC frequency in LPS-treated mice. mEPSCs were recorded in hippocampal CA1 pyramidal neurons from these four groups. Similar to the results in Figure 2, treatment with LPS alone (group III vs. I) increased mEPSC frequency (Figure 4A,B). Metformin administration in LPS-treated mice (group IV vs. III) reduced the mEPSC frequency to that of group I (Figure 4A,B). mEPSC amplitudes were not altered between these four groups. These results suggest that metformin rescues the increased mEPSC frequency in LPS-induced depression mice. 

### 3.5. Metformin Reduces Presynaptic Glutamate Release in LPS-Induced Depression Mouse Model 

To determine if LPS and metformin change the intrinsic excitability of pyramidal neurons in the hippocampus, we observed the neuronal firing response upon different depolarizing current injections in the current clamp mode. As shown in Figure S3, there was not any change in the number of action potentials (APs) between the four different groups, which suggests neither LPS nor metformin affects the excitability of hippocampal CA1 pyramidal neurons.

**Figure 5:**
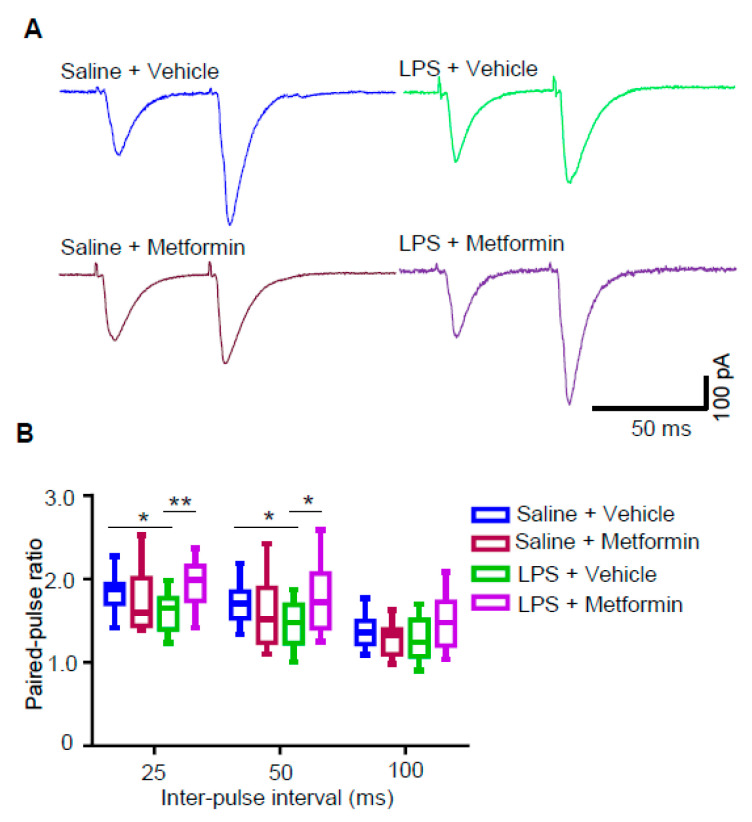
Metformin reduces presynaptic glutamate release in LPS-induced depression mouse model. (**A**) Representative traces of paired-pulse ratio (PPR) in CA1 pyramidal neurons from mice of four different groups: saline + vehicle (blue), saline + metformin (brown), LPS + vehicle (green) and LPS + metformin (purple). (**B**) PPRs plotted against interstimulus intervals (n = 13–14 neurons from 3 mice for each group; two-way ANOVA, * *p* < 0.05, ** *p* < 0.005).

Next, we wondered if the change in mEPSC frequency shown in Figure 2 and Figure 4 was attributed to the altered presynaptic glutamate release. We measured EPSCs evoked by two presynaptic stimulations delivered at different intervals (i.e., paired pulses), and the fraction of EPSC2/EPSC1 amplitudes was defined as the paired-pulse ratio (PPR). As shown in Figure 5, PPRs at 25 and 50 ms intervals were decreased in mice treated with LPS alone (group III vs. I), but metformin administration in LPS-treated mice (group IV vs. III) increased the PPR to that of group I. These results suggest that glutamate release probability is increased in LPS-induced depression mice and metformin rescues the increased glutamate release.

## 4. Discussion

In this study, we demonstrated that metformin ameliorates depressive-like behaviors and glutamatergic transmission in the LPS-induced depression mouse model. First, the mice administrated with LPS showed not only increased immobility in FST and TST, representing depressive-like behaviors, but also increased mEPSC frequency in the hippocampal pyramidal neurons. Second, the increased immobility in FST and TST, as well as the increased mEPSC frequency, in LPS-treated mice was reduced after metformin administration. Third, neither LPS nor metformin treatment altered the excitability of hippocampal neurons. Presynaptic glutamate release measured by PPR increased in LPS-induced depression mice, but decreased after metformin administration. These results present evidence that metformin ameliorates depressive-like behaviors and glutamatergic neurotransmission in the LPS-induced depression mouse model.

The bacterial endotoxin LPS, a characteristic component of the outer membrane of Gram-negative bacteria, induces cytokines to activate the peripheral innate immune system acutely. Administration of LPS in rodent models induces depressive-like behaviors, like increased immobility in the FST and TST, decreased consumption of saccharin solutions, suppression of sexual behavior and attenuation of cocaine-induced place preference [36,37,41]. Some depressive-like behaviors induced by LPS can be attenuated by chronic antidepressant treatment [37]. As such, LPS-treated mice have been used as a depression model for novel antidepressant screening [42,43]. To evaluate depressive-like behaviors in mice, we adopted FST and TST in our study, which have been used widely for the preclinical screening of antidepressants. Here, we observed that LPS-treated mice exhibited increased immobility time in the FST and TST (Figure 1). However, these two tests depend on mice’s normal locomotor behavior, which we did not detect in our mice model. Some studies found that LPS administration in mice induces increased immobility in FST and TST without affecting locomotor activity [36,44,45,46]. In contrast, it is also reported that LPS treatment in mice or rats decreases locomotor activity [47,48]. Taken together, we can detect locomotor activity in our mouse model in future experiments. Other depressive-like behavioral tests, like the sucrose preference test, could also be taken further.

It has been demonstrated that LPS treatment plays roles in synaptic transmission and plasticity. Exposure of rat cortical slices to LPS induces a rapid and sustained release of glutamate [49]. LPS suppresses rat hippocampal long-term potential (LTP) in vitro [50,51] and in vivo [52]. Paired-pulse facilitation (PPF) or PPR is reduced in LPS-treated rats, which suggests that LPS increases presynaptic release probability [52]. Brain slices exposed acutely to LPS ex vivo showed enhanced excitatory synaptic transmission and neuronal excitability [53]. Reduced frequency of mEPSC in acute hippocampal slices treated with LPS was also reported [54]. Here, increased mEPSC frequency and reduced PPR (Figure 2, Figure 4 and Figure 5), suggesting increased presynaptic glutamate release, were observed in LPS-treated mice. However, no significant change in neuronal excitability was observed in LPS-treated mice (Figure S3). Different treatment methods or doses may contribute to different results among studies. Taken together, these data indicate that glutamatergic transmission is elevated in LPS-treated mice. In synapses, there are spontaneous and evoked forms of neurotransmitter release. At rest, spontaneous glutamate release without action potentials gives rise to mEPSCs, reflecting the quantal events of synaptic transmission [55]. Evoked glutamatergic transmission, reflected by an evoked EPSC (eEPSC) recording upon stimulation of fiber tracts, symbolizes synaptic transmission between two neurons. However, the properties and working mechanisms of mEPSCs and eEPSCs could be quite different [56,57,58]. Thus, it is hard to predict the change of eEPSCs induced by LPS or metformin, which could be examined in the future. 

Although metformin is the mainstay of therapy for T2DM, with a long history, it has also been indicated that metformin may have effects in some neurological disorders, like neurodevelopmental disorders, neurodegenerative diseases and neuropsychiatric disorders. In a fragile X syndrome (FXS) mouse model, i.p. injection of metformin corrected core deficits, like the social deficit, repetitive behavior, abnormal dendritic spine morphology and exaggerated long-term depression (LTD) [59]. Moreover, language, cognitive and behavioral improvements have been found in a small sample size of metformin-treated FXS patients [60,61,62]. In an Alzheimer’s disease (AD) mouse model, metformin attenuated amyloid plaque deposition and spatial memory deficit [63]. Metformin also had neuroprotective effects for dopaminergic neurons in a 1-methyl-4-phenyl-1,2,3,6-tetrahydropyridine (MPTP)-induced Parkinson’s disease (PD) mouse model [64,65]. Moreover, metformin has been found to rescue the early brain changes and abnormal behaviors in a Huntington’s disease (HD) mouse model [66].

For depression, metformin can reduce depressive symptoms in T2DM patients with depression [11]. In mice, metformin ameliorates depressive-like behaviors induced by CSDS [15] or CUMS [16]. A recent study reported that metformin administration at 100 and 200 mg/kg, but not 300 mg/kg, rescued the increased immobility in FST and TST in LPS-treated male Swiss albino mice [67]. Consistent with this result, we also found that the increased immobility time in FST and TST were reduced after administration of metformin at 200 mg/kg in LPS-treated male C57BL/6 mice (Figure 3). Not only depressive-like behaviors but also elevated glutamatergic transmission was rescued upon metformin administration in LPS-treated mice. In our study, we found that LPS-treated mice exhibited increased mEPSC frequency and reduced PPR and metformin ameliorated these deficits (Figure 2, Figure 4 and Figure 5). Interestingly, some other compounds, like galantamine and anthocyanins, which can improve the deficits induced by LPS in mice, also have antidepressant effects [68,69,70,71]. This implies that the downstream pathway of LPS may be a target for antidepressants. LPS induces cytokines (like interleukin-6 (IL-6) and tumor necrosis factor-α (TNF-α)) to activate the immune system, while metformin has anti-inflammation effects and can suppress the induction of IL-6 and TNF-α induced by LPS [72]. Many cytokines regulating neuronal excitability, synaptic transmission and plasticity are also found in the brain. The rescue of glutamatergic transmission deficits by metformin in LPS-treated mice may be attributed to the anti-inflammation effect of metformin, which needs further examination.

Aside from metformin, some other antidiabetic drugs like insulin, pioglitazone, glyburide, liraglutide, vildagliptin and exenatide are also reported to have antidepressant effects, which may be mediated by lowering the blood glucose level, attenuating oxidative stress and inflammation and modulating the hypothalamic–pituitary–adrenal axis [73]. In diabetes, metformin is used for insulin resistance. Insulin resistance is implicated in the pathophysiology and treatment of depression [74]. The insulin receptor is abundant in the brain, including the hippocampus [75]. Upon binding with insulin, the insulin receptor is activated and triggers the activation of downstream signaling cascades, like the PI3K (phosphoinositide 3-kinase)–Akt pathway and the MEK (MAPK/ERK kinase)–ERK (extracellular signal-regulated kinase) pathway [76]. Activating insulin receptors in the hippocampus contributes to the enhancement of cognition induced by insulin [77,78]. Thus, this insulin receptor pathway may also play roles in the antidepressant effects of metformin.

## 5. Conclusions

Taken together, our results suggest that metformin ameliorates depressive-like behaviors induced by LPS and the antidepressant effect of metformin may be carried out through its downregulation of glutamatergic transmission in the LPS-induced depression mouse model. Here, we provided an insight into the underlying mechanism by which metformin acts against depression.

## Supplementary Materials

The following are available online at https://www.mdpi.com/2079-7737/9/11/359/s1. Figure S1: LPS treatment does not alter gross brain size, weight, and morphology, Figure S2: Metformin treatment in the LPS-induced depression mouse model does not alter gross brain size, weight, and morphology, Figure S3: LPS and Metformin do not affect the excitability of hippocampal CA1 pyramidal neurons.
