# Supplementary material for: Metformin Ameliorates Lipopolysaccharide-Induced Depressive-Like Behaviors and Abnormal Glutamatergic Transmission
Source: Biology (Basel). 2020 Oct 26;9(11):359. doi: 10.3390/biology9110359 (PMC7692296; doi:10.3390/biology9110359)
Supplement: Supplementary file 1 [file biology-09-00359-s001.pdf]

Article

Metformin ameliorates lipopolysaccharide-induced depressive-like behaviors and abnormal glutamatergic transmission

Jiang Chen, Tian Zhou, A-Min Guo, Wen-Bing Chen, Dong Lin, Zi-Yang Liu and Erkang Fei

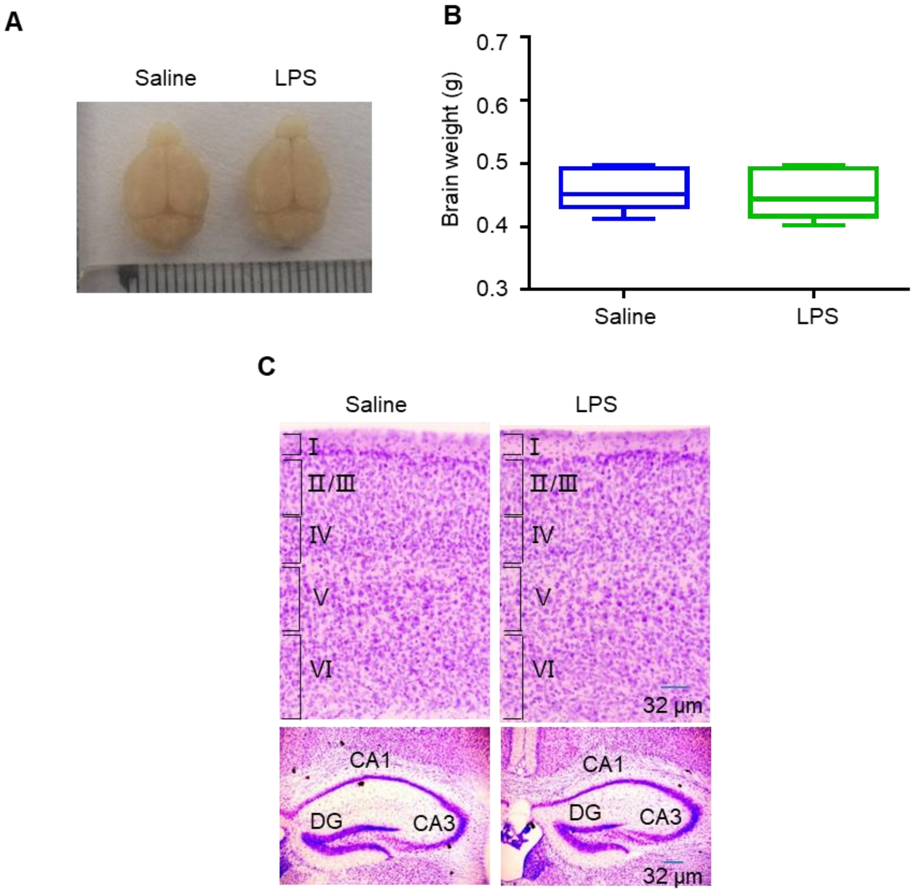

Figure S1. LPS treatment does not alter gross brain size, weight, and morphology. (**A**): Representative brain images of LPS-treated (right) and saline-treated mice (left). (**B**): Quantitative analysis of brain weight of LPS-treated and saline-treated mice. (n = 8 mice for both group; Student's t-test, p>0.05). (**C**): Gross cortex (upper panel) and hippocampus (lower panel) morphology of LPS-treated (right) and saline-treated mice (left) by Nissl staining.

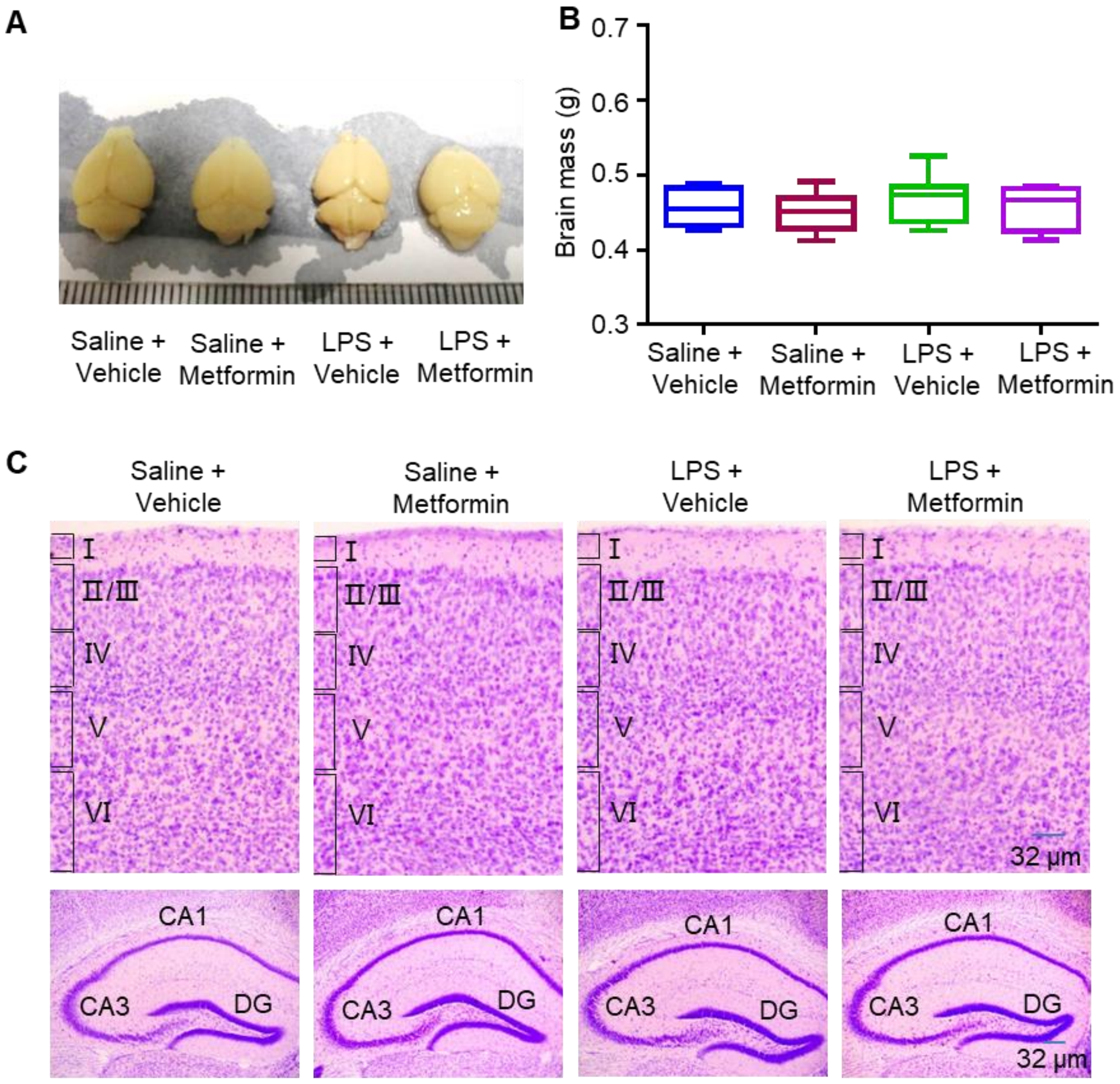

Figure S2. Metformin treatment in the LPS-induced depression mouse model does not alter gross brain size, weight, and morphology. (**A**): Representative brain images of four different treatment groups as indicated. (**B**): Quantitative analysis of brain weight of mice from four different treatment groups as indicated. (n = 8 mice for each group; one-way ANOVA, p=0.6037). (**C**): Gross cortex (upper panel) and hippocampus (lower panel) morphology of four different treatment groups as indicated.

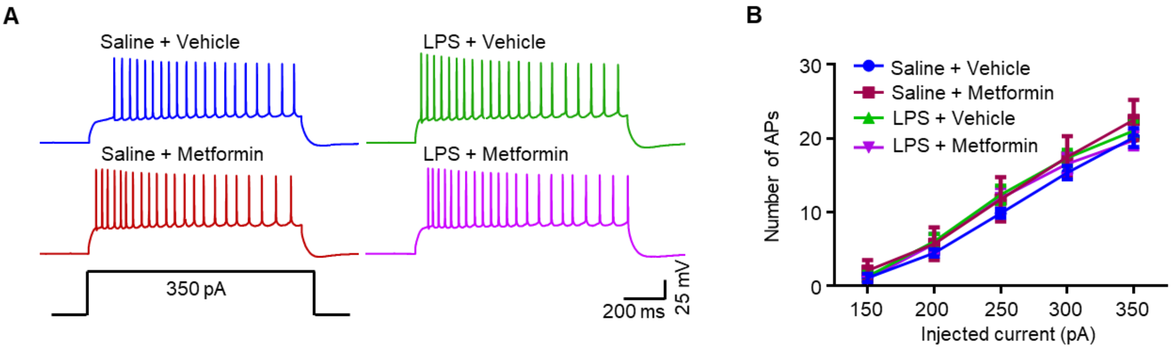

Figure S3. LPS and Metformin do not affect the excitability of hippocampal CA1 pyramidal neurons. (**A**): Representative trains of action potentials (APs) induced by a depolarizing current (1s, 350 pA) injection in Saline + Vehicle (blue), Saline + Met (brown), LPS + Vehicle (green), and LPS + Met (purple)

four different groups mice. (**B**): Number of APs induced by different depolarizing currents injection. (n = 16 neurons from 3 mice for each group; two-way ANOVA, p=0.5647).

© 2020 by the authors. Licensee MDPI, Basel, Switzerland. This article is an open access article distributed under the terms and conditions of the Creative Commons Attribution (CC BY) license (http://creativecommons.org/licenses/by/4.0/).
